# An unusual case of pleomorphic adenoma of the nasolacrimal duct

**DOI:** 10.1002/ccr3.8504

**Published:** 2024-02-12

**Authors:** Katherynn Zhang, Adrian Ivar Mendez

**Affiliations:** ^1^ Schulich School of Medicine & Dentistry Lawson Health Research Institute, Western University London Ontario Canada; ^2^ Department of Otolaryngology‐Head and Neck Surgery Western University London Ontario Canada

**Keywords:** ear, nose, and throat, oncology

## Abstract

**Key Clinical Message:**

Pleomorphic adenoma is a common epithelial tumor but is unusual to involve the nasolacrimal duct and lacrimal sac. Current reported cases are sparse but may be helpful for delineating patterns of malignant transformation in the future.

**Abstract:**

This is a report of a 66‐year‐old patient with a pleomorphic adenoma involving the nasolacrimal duct and lacrimal sac, which is an unusual location for these tumors. To our knowledge, there are scarce publications in the current literature of similar cases.

## Graphical abstract

Left nasolacrimal duct pleomorphic adenoma.

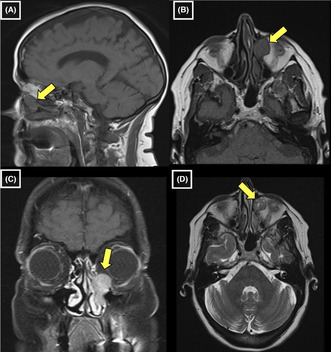

## 1 INTRODUCTION

Pleomorphic adenoma (PA), also called benign mixed tumor, is the most common type of epithelial tumor in the salivary gland and typically occurs among adults (female to male ratio of 2:1).^1^ Although PA represents most of the cases of salivary gland tumors, its incidence is low and especially unusual involving the nasolacrimal duct and lacrimal sac.^2^, ^3^ PA is correlated with slow growth with majority of common clinical symptoms present for over a year.^4^

Common clinical features from published reviews include proptosis and ocular globe displacement.^4^, ^5^ Less common symptoms include sensory loss and pain while atypical symptoms may be inflammatory in nature, including orbital cellulitis.^4^, ^5^ Clinicopathologic review of patients with lacrimal tumors with radiologic correlation described bone remodeling for well‐delineated benign masses (e.g., PA) but bone invasion and destruction in malignant lesions (e.g., adenoid cystic carcinoma).^5^ Benign lacrimal epithelial neoplasia to consider beyond PA include myoepithelioma and oncocytoma, both of which are rare in reported literature.^6^ Differential considerations of malignant neoplasms include adenoid cystic carcinoma, carcinoma ex‐pleomorphic adenoma, and mucoepidermoid carcinoma.^5^, ^6^ Pleomorphic adenoma in the naso‐orbital region are typically managed with dacryocystorhinostomy to remove the mass.^2^ Use of radiation as a treatment modality is rare for PA but possible if lesions are inoperable or residual.^4^

## 2 CASE

**FIGURE 1:**
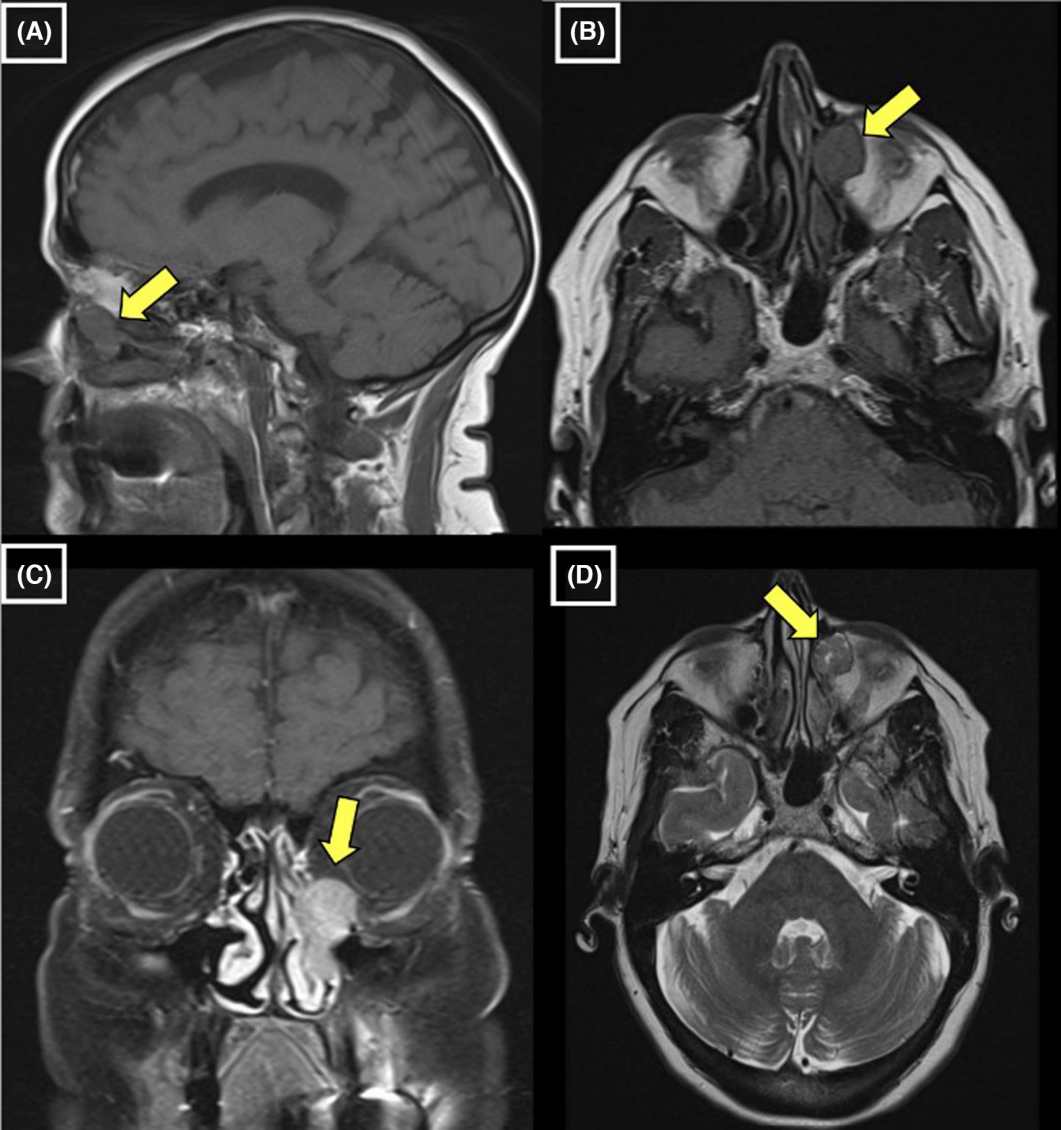
(A) T1 Sagittal view of a lobular/frondlike soft tissue mass centered within, and expanding, the left nasolacrimal duct measuring approximately 1.5 × 1.4 × 2.5 cm [yellow arrow]. (B) T1 Axial view of the tumor [yellow arrow]. (C, D) T1 coronal and T2 axial images showing near homogeneous enhancement of the lesion following contrast administration [yellow arrow].

We report the case of a 66‐year‐old woman who was referred to the Head and Neck Surgery Department for symptoms related to a salivary gland tumor. Five years prior to the referral, she began experiencing blockage in her left tear duct, for which she underwent a left dacryocystorhinostomy in 2017. This past year, the patient had began experiencing nasal obstruction, tearing and crusting of her left eyelid, and loss of sensation at the tip of her tongue. Initial investigations showed an incidental left maxillary/infraorbital mass on a magnetic resonance in 2021 (Figure 1A–D). She subsequently underwent a biopsy of the mass which proved inconclusive. The mass was considered to be a nasolacrimal neoplasm of salivary gland origin with no specified subtype at this time due to inconclusive pathology and limited sample size. Differential considerations included possible squamous cell papilloma versus squamous cell carcinoma, among other considerations. The patient consented for surgical removal of the mass as part of the suggested management plan given she was symptomatic from the mass effect on surrounding structures.

A left partial maxillectomy was performed to remove the tumor from the inferior orbital rim and medial orbit. As the tumor appeared to be involving the nasolacrimal duct, a left dacryocystorhinostomy was also performed. Intraoperative findings revealed a lesion within the left inferior medial canthus of the left eye abutting the orbital rim and the orbital muscles. This was in close proximity to the junction of the left nasal bone with the maxillary bone. The lesion did not have a firm capsule; it did not appear to be invading surrounding structures but appeared to be pushing on the surrounding structures instead. Left nasal mucosal and left infraorbital samples from the procedure were sent for pathologicanalysis. No neoplastic tissue was identified from the nasal component of the mass. The left infraorbital portion of the mass showed features consistent with benign salivary gland neoplasm and pleomorphic adenoma. The patient recovered well postoperatively, without major complications, and was discharged on postoperative Day 1.

## 3 DISCUSSION

The few studies that exist in the literature regarding lacrimal tumors generally report on disease recurrence and malignant transformation. For recurrence, one study reported three patients with disease relapse (23, 25, and 30 years post resection) from a group of 118 lacrimal tumors, 48% of whom had PA.^6^ However, other contradictory evidence reported up to a 10%–40% rate of recurrence after treatment.^2^ There is a reported 10% chance of malignant transformation (carcinoma ex‐pleomorphic adenoma) in 20 years and 20% in 30 years for lacrimal PA.^4^ To our knowledge, no clear evidence has been reported yet for nasolacrimal duct PA specifically. Currently, the literature reports that PA may be associated with the PLAG1 gene translocation t (5;8) (p13;q12).^7^ However, there are no specific markers currently to predict malignant transformation.^4^

At 1‐month postoperative follow‐up, the patient endorsed bloody mucous and paresthesia in the left side of her nose, with no evidence of recurrence. The patient reported having a bladder infection following her operation, which has since resolved with antibiotic treatment. No other symptoms were reported, and healing was uncomplicated. At her 3‐month follow‐up, the patient had been healing well with no facial swelling, dysphonia, dysphagia, or abnormal changes in vision. Her nasolacrimal stent was removed with no complications. At her final 10‐month follow‐up, the patient was well overall without major complications, lymphadenopathy, facial weakness, or abnormalities on nasopharyngeal scope. She did not have any major concerns for facial weakness or suspicions for recurrence.

## 4 CONCLUSION

In conclusion, pleomorphic adenoma involving the nasolacrimal duct and lacrimal sac is an unusual entity. Its standard treatment is surgical resection, and to our knowledge, there is no clear, consistent evidence in the literature regarding malignant transformation involving the nasolacrimal duct. Our reported case of a 66‐year‐old female patient recovered well over 10 months after her left partial maxillectomy and left dacryocystorhinostomy with no known major complications or recurrence.

## AUTHOR CONTRIBUTIONS

**Katherynn Zhang:** Writing – original draft; writing – review and editing. **Adrian Ivar Mendez:** Conceptualization; formal analysis; investigation; methodology; project administration; resources; supervision; writing – original draft; writing – review and editing.

## FUNDING INFORMATION

The authors have no financial disclosures to declare.

## CONFLICT OF INTEREST STATEMENT

The authors have no conflict of interest to declare.

## ETHICS STATEMENT

Patient consent was obtained as part of this case report.

## CONSENT

Written informed consent was obtained from the patient to publish this report in accordance with the journal's patient consent policy.

## Data Availability

Data sharing is not applicable to this article as no new data were created or analyzed in this study.
